# Dynamic transcriptome profiles of skeletal muscle tissue across 11 developmental stages for both Tongcheng and Yorkshire pigs

**DOI:** 10.1186/s12864-015-1580-7

**Published:** 2015-05-12

**Authors:** Yuqiang Zhao, Ji Li, Huijing Liu, Yu Xi, Ming Xue, Wanghong Liu, Zhenhua Zhuang, Minggang Lei

**Affiliations:** Key Laboratory of Agricultural Animal Genetics, Breeding, and Reproduction of Ministry of Education and Key Laboratory of Swine Genetics and Breeding of Ministry of Agriculture, Huazhong Agricultural University, Wuhan, PR China; National Animal Husbandry Services Ministry of Agriculture, Beijing, PR China; BGI-Shenzhen, Shenzhen, PR China

**Keywords:** Tongcheng and Yorkshire pigs, Skeletal muscle, Bioinformatic transcriptome, Histology and histochemistry, qPCR

## Abstract

**Background:**

The growth and development of skeletal muscle directly impacts the quantity and quality of pork production. Chinese indigenous pig breeds and exotic species vary greatly in terms of muscle production and performance traits. We present transcriptome profiles of 110 skeletal muscle samples from Tongcheng (TC) and Yorkshire (YK) pigs at 11 developmental periods (30, 40, 55, 63, 70, 90, and 105 days of gestation, and 0, 1, 3, and 5 weeks of age) using digital gene expression on Solexa/Illumina’s Genome Analyzer platform to investigate the differences in prenatal and postnatal skeletal muscle between the two breeds.

**Results:**

Muscle morphological changes indicate the importance of primary fiber formation from 30 to 40 dpc (days post coitus), and secondary fiber formation from 55 to 70 dpc. We screened 4,331 differentially expressed genes in TC and 2,259 in YK (log_2_ ratio >1 and probability >0.7). Cluster analysis showed different gene expression patterns between TC and YK pigs. The transcripts were annotated in terms of Gene Ontology related to muscle development. We found that the genes *CXCL10*, *EIF2B5*, *PSMA6*, *FBXO32*, and *LOC100622249* played vital roles in the muscle regulatory networks in the TC breed, whereas the genes *SGCD*, *ENG*, *THBD*, *AQP4*, and *BTG2* played dominant roles in the YK breed. These genes showed breed-specific and development-dependent differential expression patterns. Furthermore, 984 genes were identified in myogenesis. A heat map showed that significantly enriched pathways (FDR <0.05) had stage-specific functional regulatory mechanisms. Finally, the differentially expressed genes from our sequencing results were confirmed by real-time quantitative polymerase chain reaction.

**Conclusions:**

This study detected many functional genes and showed differences in the molecular mechanisms of skeletal muscle development between TC and YK pigs. TC pigs showed slower muscle growth and more complicated genetic regulation than YK pigs. Many differentially expressed genes showed breed-specific expression patterns. Our data provide a better understanding of skeletal muscle developmental differences and valuable information for improving pork quality.

**Electronic supplementary material:**

The online version of this article (doi:10.1186/s12864-015-1580-7) contains supplementary material, which is available to authorized users.

## Background

The development of skeletal muscle affects meat production and growth rate in pigs. The genetic mechanisms controlling these traits are being uncovered [1,2]. In addition, the selection of meat quality based on molecular regulation is important for livestock breeding, and there have been many studies on skeletal muscle in different pig breeds [3-8]. The total number of fibers is constant in the postnatal stage, and postnatal muscle growth is through hypertrophy of myofibers, or conversion of myofiber types [9,10]. Primary and secondary fibers emerge at distinct embryonic stages during the skeletal muscle development of pigs [11]. Primary myofiber formation occurs in the first wave of fiber generation, from about 35 days of gestation until around 60 days. Secondary myofibers appear at around 50–60 days based on the template of the primary myofiber surface. The third wave involves the process of transition between slow-oxidative and fast-glycolytic fiber types, which occurs from birth to 60 days of age [9,12,13]. Muscle maturation is completed in the early postnatal period [14]. Therefore, we chose the critical skeletal muscle developmental stages across the prenatal to postnatal periods (30, 40, 55, 63, 70, 90, and 105 days of gestation, and 0, 1, 3, and 5 weeks of age) for studying the regulatory mechanisms and expanding the molecular genetics of muscle fiber development.

Many genes or factors participate in the process of muscle fiber formation, and muscle development is under complex genetic regulation. It is known that the muscle regulatory factor (*MRF*) and myocyte-specific enhancer binding factor 2 (*Mef2*) gene families play critical roles in regulating muscle fiber development. *MRF* genes are muscle-specific transcription factors, and induce myoblast proliferation or fusion differentiation during distinct stages of myogenesis [15]. *Mef2* regulates muscle-specific transcription during myogenesis and is activated during the development of the muscle [16,17]. However, knowledge of the functional genetics of myogenesis is currently insufficient and incomprehensive. Monitoring the transcriptional profiling from the prenatal to postnatal developmental stages in pigs will act as an animal model that will not only uncover the regulatory mechanisms behind factors affecting muscle development [18], but will also benefit the understanding of diseases affecting human muscle and muscular atrophy [19]. With biotechnological advances in gene expression profiling, we have the ability to study large-scale differential regulation of genes genome wide, and to measure the transcriptional responses to certain complex biological conditions [20]. Previous studies have presented expression analysis of myogenesis using microarrays [21,22], long SAGE [23], and differential display RT (reverse transcription)-polymerase chain reaction (PCR) [24]. Digital gene expression technology is sensitive for the detection of low-abundant transcripts and small changes in gene expression [25], and has been applied extensively to compare the differences in transcription profiles of different tissues or breeds [15,26]. We present a panorama of transcriptome-wide longissimus dorsi muscle development using digital gene expression with Solexa/Illumina’s Genome Analyzer platform to study the variation between the dynamic transcriptome profiles of Tongcheng (TC) and Yorkshire (YK) pig breeds.

Previous studies have demonstrated differences in gene expression in pig longissimus dorsi muscle during fetal development, and some have shown breed-specific expression profiles [8,15,23,27-29]. Indeed, differences exist in muscle traits such as muscularity and muscle fiber type; muscle characteristics are different between pig breeds, and molecular biomarkers have been reported [30]. However, the molecular genetic mechanisms of these breed-specific differences remain unclear, in terms of meat quality and visual phenotype. In this study, we selected the TC pig, a characteristic indigenous pig breed from the Hubei Province of central China with slow growth and prolific reproduction [31], and the YK pig, which is characterized by fast growth, low back fat, and a high lean meat percentage, to survey the differences in transcription. Previous studies have shown that indigenous swine breeds have slower growth rates and less lean meat percentages than exotic pigs [7,15,23]. Our study opens up the scope of knowledge of muscle-specific regulation and the microscopic molecular mechanisms. Our objective was to analyze the biological information regarding the transcriptional profiles of longissimus muscle between the two breeds and to further identify key gene regulatory networks and breed-specific pathways affecting skeletal muscle development in the pig.

## Results

### Histological section and fiber size

We evaluated the morphological differences of skeletal muscle development between TC and YK pigs, from the histological appearance through the prenatal and postnatal stages. Histological cross-section (Additional file 1: Figure S1) showed that myoblasts developed quickly from 30 dpc (days post coitus), but there was no differentiation into primary fibers in either pig breed at 30 dpc, and the number and density of myoblasts in TC was more than that in YK. The myoblasts differentiated gradually, with primary fibers emerging from 30 to 40 dpc, and could be seen in the histological section to be fully formed at 40 dpc. Primary fibers continued to expand and enlarge until 63 dpc. Meanwhile, the primary fiber bundles were separated by the connective tissues. Secondary fibers appeared at 55 dpc in YK pigs and formed until 63 dpc, but they emerged later in TC pigs. Secondary fibers could be seen clearly around the primary fibers at 63 dpc, and the secondary fibers increased gradually until 90 dpc. The fiber bundles fused at around 90 dpc and appeared to form into the myotubes at 105 dpc; the primary and secondary fibers were not distinguishable at this period. The intramuscular fat texture showed differences in the histological section in the latter period between TC and YK pigs. These differences are associated with the different development of fat deposits in these two breeds. The muscle fibers grew rapidly, and finally the muscular tube formed and constituted the muscle mass. The phenotypes and sizes of the muscle fibers still varied based on the cross-sectional areas during the postnatal development stages (1, 3, and 5 weeks post natum (wpn) for both TC and YK pigs).

### Solexa sequencing and tag mapping

After filtering adaptor tags, empty tags, low-quality tags, and one-copy tags from the raw data, 61,829,793 total clean tags were obtained in the TC breed, including 1,726,839 distinctly clean tags. 63,122,631 total clean tags and 1,684,960 distinctly clean tags were obtained in the YK pig (Additional file 2: Table S1). The total clean tags accounted for 93.6% and 94.3% of the raw tags in TC and YK, respectively. The number of unknown distinct tags was 32,884 and 33,141, on average, in each sample from TC and YK pigs. Moreover, the heterogeneity and redundancy of the mRNA were confirmed, and the results showed that the high copy number clean tags (with a copy number of more than 100) accounted for 76.5% of the total clean tags in TC and 77.5% in YK, while the low copy number clean tags (with a copy number of less than 2) accounted for less than 1% of the total clean tags in both breeds. Additional file 3: Figure S2 illustrates the distribution of total clean tags and distinct clean tags of TC and YK. The type of distinct tags were gradually stable, with the total number of sequence tags rising according to saturation analysis, and the percentage of genes identified was no longer increasing when the library size approached 3 million (Additional file 4: Figure S3). The number of tags mapping to genes was 283,853 and 283,869 in TC and YK, and the number of genes with unambiguous tag mapping was 200,320 and 199,991. The number of unambiguous tags mapping to genes accounted for 70.6% of all tags mapping to genes, and 58.9% of the total clean tags for the TC breed, in contrast to 70.4% and 60.0% for the YK breed (Additional file 2: Table S1).

### Differential gene expression analysis and validation of Solexa sequencing data

**Figure 1:**
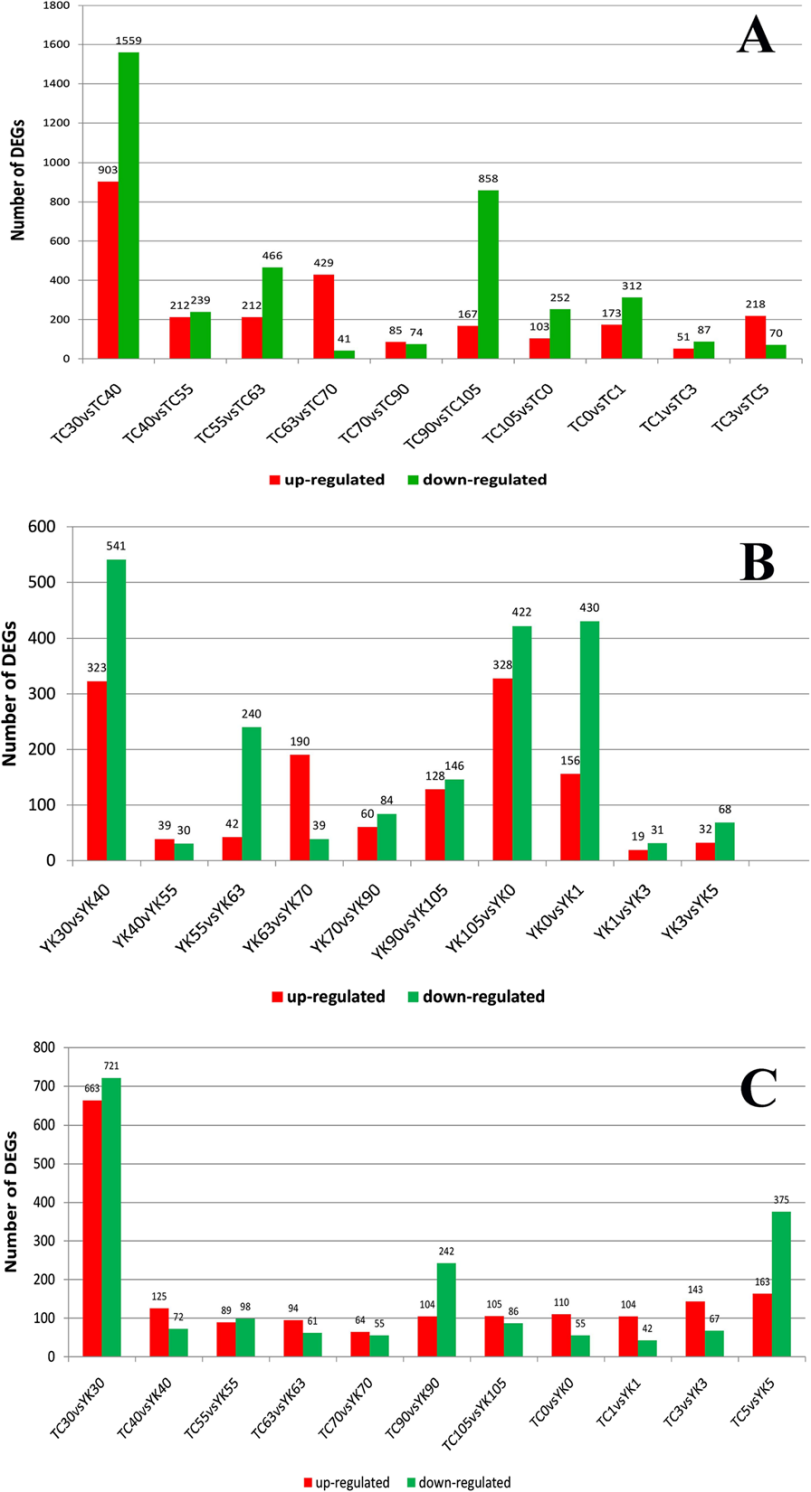
Number of DE genes between the comparison libraries. TC: Tongcheng, YK: Yorkshire. Before birth: 30, 40, 55, 63, 70, 90, and 105 days post-coitus. After birth: 0, 1, 3, and 5 weeks post-natum. **A**: Tongcheng pigs, **B**: Yorkshire pigs, **C**: the identical period comparison between TC and YK pigs.

In this study, differentially expressed (DE) genes at the 11 time points of skeletal muscle development of TC and YK pigs were investigated through transcriptome-wide Solexa sequencing, and analyzed using the NOIseq method. In the TC libraries, a total of 4,331 genes were found to be DE; of these, 2,553 genes were upregulated and 3,958 genes were downregulated in 10 comparison libraries (Additional file 5: Table S2 and Figure 1). In the YK libraries, a total of 2,259 DE genes were identified, with a total of 1,503 upregulated and 2,033 downregulated genes in 10 comparison libraries during different muscle development stages. These results suggest that the number of DE genes changed significantly at 30–40 dpc, 55–63 dpc, and 90–105 dpc in the prenatal muscle development stages, and at 3–5 wpn in the postnatal stages.

**Figure 2:**
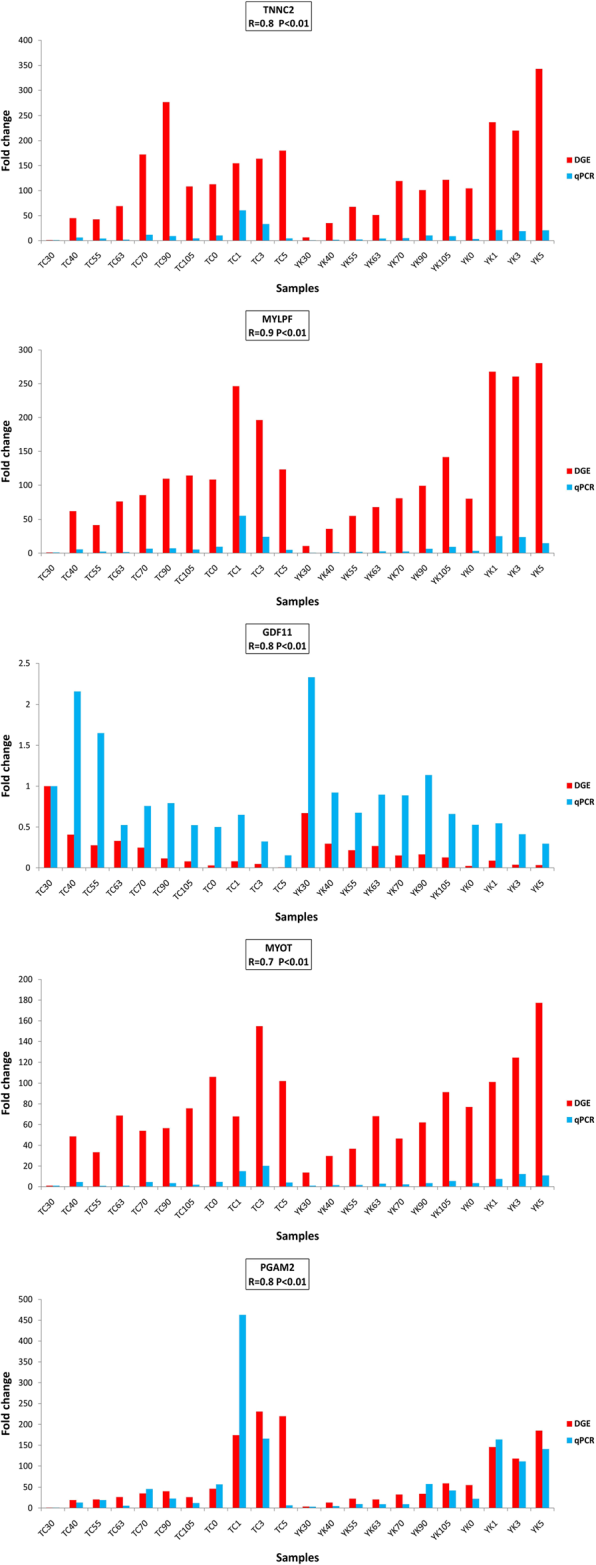
Validation of Solexa sequencing data with qPCR. The vertical axis indicates the fold change of transcript abundance. The horizontal axis indicates the samples for TC and YK pigs. The r-value shows the Spearman’s correlation between the two methods.

Five genes were chosen randomly for the purpose of validating the results of the Solexa sequencing using real-time quantitative PCR (qPCR). *TNNC2* (Troponin C Type 2) is a key gene encoding a protein that regulates striated muscle contraction [32]. *GDF11* (Growth Differentiation Factor 11) regulates cell growth and differentiation in both embryonic and adult tissues, and is related to skeletal muscle age [33]. *MYOT* (myotilin) encodes a cytoskeletal protein that is involved in myofibril assembly and actin binding in the muscle tissue [34]. *PGAM2* (Phosphoglycerate Mutase 2) is a muscle-specific phosphoglycerate mutase and is involved in the glycolysis pathway for myoblast fusion [35]. *MYLPF* (Myosin Light Chain, Phosphorylatable, Fast Skeletal Muscle) is related to a structural constituent of muscle and calcium ion binding [36]. In our study, *RAD23 Homolog B* was used as the reference gene because it showed consistent expression. The data and Spearman’s correlation coefficient analysis suggest that the Solexa sequencing had a highly significant correlation with real-time qPCR (Figure 2), and the expression patterns of these genes were consistent between the two methods.

### Cluster analysis of DE genes

**Figure 3:**
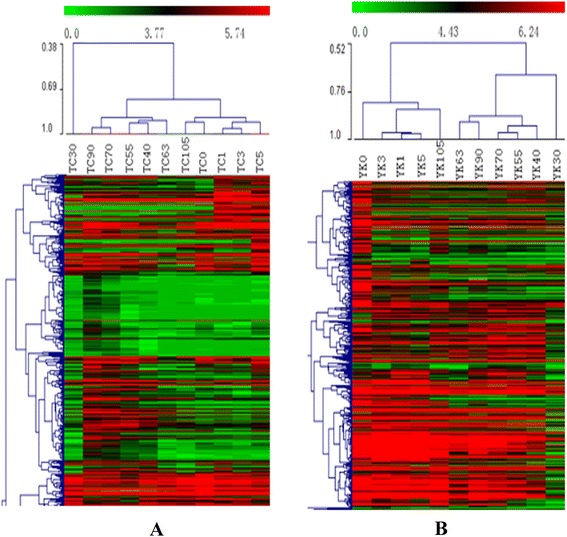
Hierarchical clustering analysis for all the DE genes in TC and YK pig breeds, respectively. **A**: TC (Tongcheng), **B**: YK (Yorkshire).

Gene expression patterns at the scale of the transcriptome were measured by systematic cluster analysis, to explore the similarities and compare the relationship between the different libraries in TC and YK pigs. The results of hierarchical clustering indicate that the gene expression similarity could be classified into distinct groups (Figure 3). TC40, TC55, and TC63 were clustered together, and TC70 and TC90 were of the same class; while YK55 and YK70 had similar expression patterns, and YK63 and YK90 were clustered into another class in the prenatal stage owing to the similarity of gene expression. The gene expression patterns of 1, 3, and 5 weeks of age were grouped together in both breeds.

### GO analysis of DE genes

**Figure 4:**
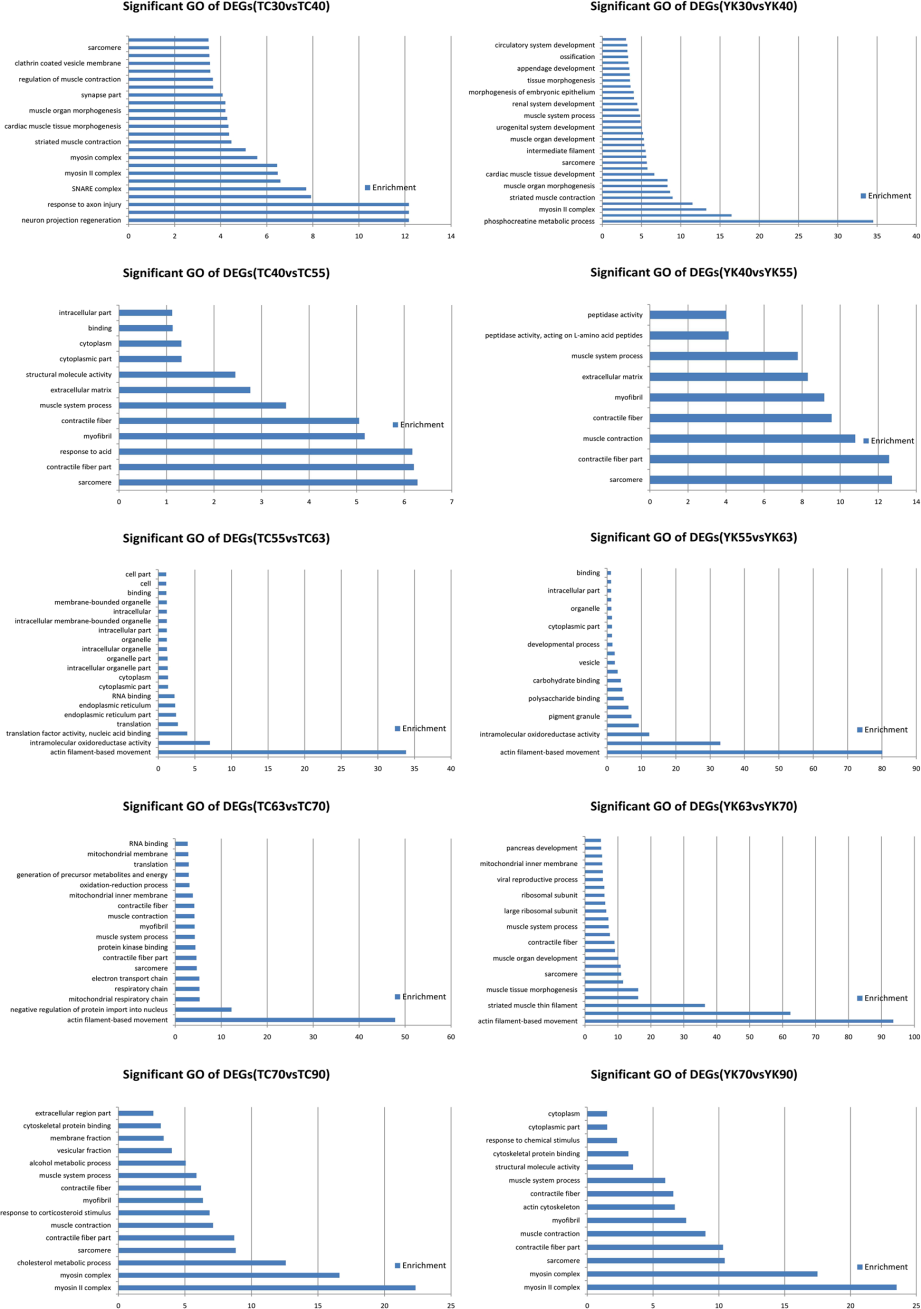
Significant GO terms of DE genes (DEGs) of each comparison library in TC and YK.

The screened DE genes were classified by gene ontology (GO) based on their biological process and function in TC and YK pigs. The GO annotation results are presented in Additional file 6: Table S3 and Additional file 7: Table S4. Significant GO categories were selected (FDR <0.05), and showed different extents of enrichment (Figure 4, Additional file 8: Figure S4). GO analysis showed that biological processes related to muscle development were mainly enriched in different stages for both breeds; these genes were associated with GO terms including myofibril, sarcomere, and myosin complex, which are involved in muscle component and structure. Muscle tissue morphogenesis and development, cell differentiation and development, regulation of muscle contraction and developmental process were enriched significantly in early embryo development. More DE genes were involved with functional processes in the postnatal stages, which were distributed between the proteasome complex, actin filament-based movement, intramolecular transferase activity, and lactate dehydrogenase activity. Meanwhile, the GO terms of embryonic organ morphogenesis, neurogenesis, and development were also associated with many DE genes. Although the GO annotation and biological process categories were similar in many aspects involved in embryo and myogenesis development between TC and YK pigs, the amount of DE genes involved in certain biological processes were quite different between the two breeds.

### Pathway enrichment analysis between TC and YK

**Figure 5:**
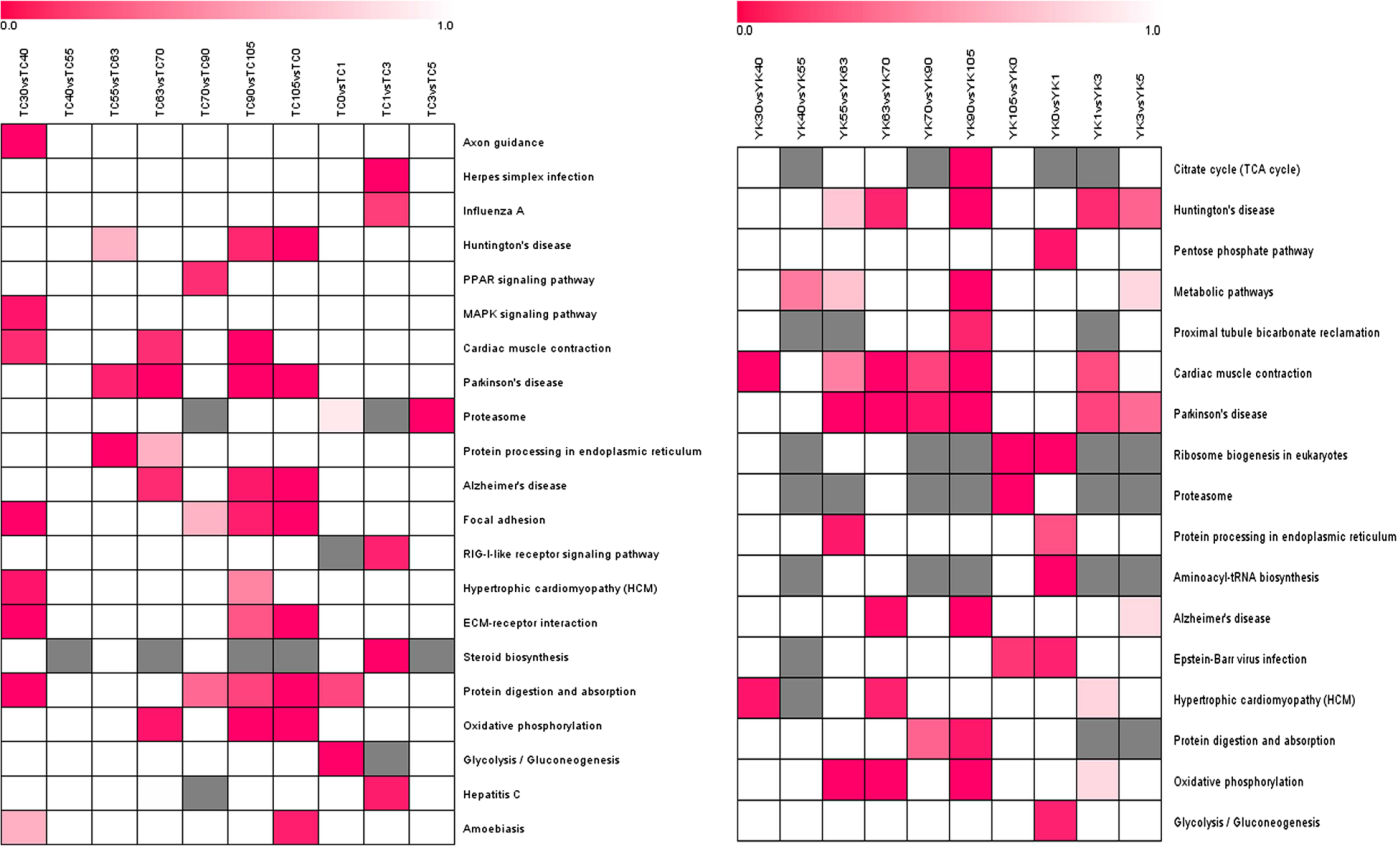
Different functional category pathway enrichment analysis of Tongcheng and Yorkshire pigs according to the FDR value among the comparison libraries using the Genesis software (FDR <0.05). Red: significant enrichment; white: non-significant; gray: not detected.

To identify the biological pathways that were involved in muscle development, we mapped the DE genes (4,331 in TC and 2,259 in YK pigs) to the reference canonical pathways in the KEGG database (Additional file 9: Table S5). In addition, we also separately gained pathways from 10 different comparison libraries in TC and YK pigs (Additional file 10: Table S6, Additional file 11: Table S7). In the top 10 significantly enriched pathways, cardiac muscle contraction, Parkinson’s disease, and citrate cycle (TCA cycle) are shared between TC and YK pigs (Tables 1 and 2). Focal adhesion, protein digestion and absorption, GABAergic synapse, axon guidance, ECM-receptor interaction, MAPK signaling pathway, arginine, and proline metabolism were found to be more associated with the DE genes in TC pigs, while oxidative phosphorylation, Huntington’s disease, ribosome biogenesis in eukaryotes, metabolic pathways, Alzheimer’s disease, glycolysis/gluconeogenesis, and proteasome are closely related to YK pigs. Furthermore, the heat map results show that different pathways have distinct functional category enrichments between the 10 comparison libraries of the two breeds (Figure 5). For instance, axon guidance shows significant enrichment in the early periods of muscle development in TC pigs, but not in YK pigs. The citrate cycle (TCA cycle) performs important roles close to birth in YK pigs, but not in TC pigs. These differences provide some cues to survey the spatial and temporal expression of genes.

**Table 1:** **Top 10 functional enrichment pathways in Tongcheng pigs**

**Path-ID**	**Path name**	**DEGs**	**FDR**	**Enrichment**
ko04260	Cardiac muscle contraction	41	0.001	2.4
ko05012	Parkinson’s disease	66	0.002	1.9
ko04510	Focal adhesion	117	0.004	1.5
ko04974	Protein digestion and absorption	55	0.016	1.8
ko04727	GABAergic synapse	39	0.017	2.1
ko04360	Axon guidance	79	0.061	1.6
ko04512	ECM-receptor interaction	63	0.099	1.6
ko04010	MAPK signaling pathway	99	0.228	1.4
ko00020	Citrate cycle (TCA cycle)	16	0.397	2.6
ko00330	Arginine and proline metabolism	27	0.454	2.0

**Table 2:** **Top 10 functional enrichment pathways in Yorkshire pigs**

**Path-ID**	**Path name**	**DEGs**	**FDR**	**Enrichment**
ko05012	Parkinson’s disease	59	1.96E-09	3.0
ko04260	Cardiac muscle contraction	33	4.32E-06	3.4
ko00190	Oxidative phosphorylation	47	4.13E-05	2.5
ko05016	Huntington’s disease	55	3.06E-03	1.9
ko03008	Ribosome biogenesis in eukaryotes	28	6.55E-03	2.5
ko01100	Metabolic pathways	206	7.54E-03	1.3
ko05010	Alzheimer’s disease	50	1.98E-02	1.8
ko00010	Glycolysis/Gluconeogenesis	22	3.10E-02	2.6
ko00020	Citrate cycle (TCA cycle)	13	4.25E-02	3.7
ko03050	Proteasome	15	5.52E-02	3.2

## Discussion

Previous studies have investigated the differences in gene expression patterns or molecular genetic mechanisms of skeletal muscle development between characteristically different pigs [5,8,37,38]. Our studies will be helpful to explore and elucidate the control mechanisms for muscle development and growth on a genome-wide scale. Moreover, TC pigs, a typical local breed in central China, have a developmental period with three time points for skeletal muscle [23], which may limit studies on muscle development; comparative genomics could uncover the determining factors for the genetic basis of biological functions [39]. The pig genomic sequence is very similar to the human genome, even though pigs and humans are divergent species [40]. Transcriptional profiling in our study may provide a reference for studies on human muscle tissue development and dysfunction.

Muscle fiber development occurs in two waves, around 35 and 65 dpc, which involve the formation of primary and secondary fibers [11,28]. In our study, primary and secondary fibers formed at approximately 40 and 63 dpc (Additional file 1: Figure S1), and the muscle fiber diameter at birth was larger in the YK pig than the TC pig. Previous studies have also shown that the muscle fiber diameter of a Chinese indigenous pig (Langtang, LT) is smaller than an exotic pig (Landrace, LR), and that myogenesis starts earlier but progresses more slowly in LT pigs than in LR pigs [15]. Likewise, Duroc and Pietrain pig breeds are also extremes, with myogenesis initiating earlier in Duroc pigs than in Pietrain pigs [8]. The number of myoblasts in early TC embryos was more than that of early YK embryos, which may be the result of the proliferation and differentiation of myogenic precursor cells beginning earlier and developing more intensely in early TC embryos than in early YK embryos. It is known that the number of muscle fibers determines the capacity for postnatal muscle fiber growth and hypertrophy. Importantly, postnatal muscle fiber hypertrophy is inversely correlated with the total number of fibers in the muscle [41], which may be a reason for the slower growth rate and lower lean meat content for TC pigs than for conventional western pig breeds [42]. In the histological cross-sections, muscle fiber development and differentiation were slower for TC pigs than for YK pigs. The intramuscular fat texture showed differences in the later period of muscle development between YK and TC, which may be the consequence of different expression profiles of fatty acid metabolism in the two extreme muscularity breeds [8].

Gene expression profiles can be investigated easily and extensively using RNA sequencing technology, with the results of these studies showing high levels of reproducibility in both technical and biological replicates [43,44]. To investigate the transcription profiles of TC and YK pigs reliably and comprehensively, we used five muscle samples at each stage, and evaluated the sequencing quality of each library of 110 samples (Additional file 4: Figure S3). As large and comprehensive bio-information resources were obtained, transcripts at low abundance and novel transcripts could also be detected. The distributions of total clean tags and distinct clean tags suggest that we obtained high-quality sequencing; and there were a high proportion of lower copy number tags (Additional file 3: Figure S2), which showed many genes expressed at a low level. We validated our results using real-time qPCR, which demonstrated that the quality of the Solexa sequencing is reliable and trustworthy. Moreover, qPCR measures a single specific gene, while Solexa sequencing involves massive parallel sequencing, and the abundance of the transcript can be affected by the test method and mRNA samples storage.

More DE genes were detected in the early period of muscle development (Figure 1), which reflects that the differences in the expression of muscle development genes are more intense in the period of primary fiber formation than in the period of secondary fiber formation [27]. There were 2,462 DE genes in TC pigs and 864 DE genes in YK pigs that were involved in myoblast differentiation at this stage, which indicates that TC pigs at the early stage have a more complex regulatory mechanism of initiation. In addition, cluster analysis showed that there were differences in expression patterns between TC and YK pigs, and that the gene expression patterns at 1, 3, and 5 wpn were more similar in YK than TC pigs (Figure 3). 105 dpc and 0 wpn (natal day) were more similar in TC pigs than YK pigs; however, 30 dpc was an exception. The differences at the prenatal stage were greater than the postnatal differences in the two breeds overall. These results show that muscle morphological cross-sections clearly varied from 40 to 105 dpc (Additional file 1: Figure S1), and that the primary and secondary fibers were forming and changing during these stages, which is important for the growth and development of muscle fibers.

**Figure 6:**
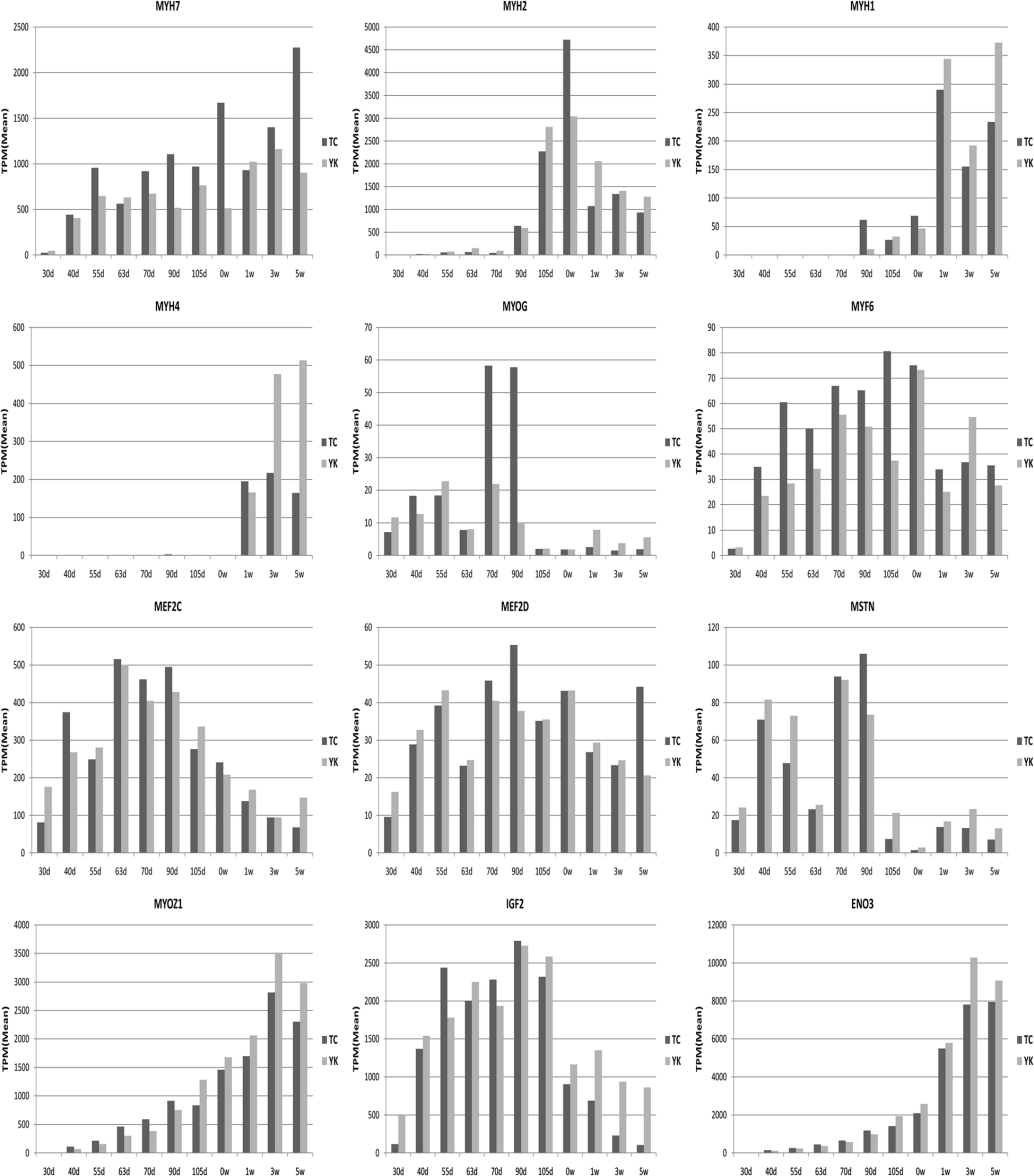
Gene expression patterns related to muscle fiber development. The vertical axis indicates the normalized gene expression level in each stage on average. The horizontal axis indicates the different developmental periods in TC and YK pigs; d indicates days prenatal and w indicates weeks of age.

Myosin heavy chain (MyHC) composition determines the type of muscle fiber and its speed-related contractile properties, and *MyHC* isoforms show different expression patterns in adult rodent muscle fibers [45,46]; fast muscles have a higher content of MyHC-IIb (encoded by the *myh4* gene) and MyHC-IIx (*myh1*) fibers, which have faster contractile speeds; while muscles with lower contractile speeds generally have a high content of MyHC-I (*myh7*) and MyHC-IIa (*myh2*) fibers [47]. *MyHC* isoforms are the primary factor determining the speed-related contractile properties of muscle fibers. Our data analysis shows that *myh1* and *myh4* expression levels were higher after birth, and higher with aging in YK pigs than in TC pigs (Figure 6). This indicates that, in the postnatal stage, the proportion of fast-glycolytic fiber types are varied and increased, and the muscle locomotor system has faster contractile speeds. *Myh2* showed a peak at birth, and *myh7* was expressed highly before and after birth with a higher level in TC pigs than YK pigs. This result indicates that TC pigs have more MyHC-I fiber, while YK pigs have more MyHC-IIb fiber.

We further analyzed the gene expression patterns related to muscle fiber development (Figure 6). *MyoG* and *MYF6* are myogenic differentiation factors [48] that participate in myoblast fusion and differentiation [27,49]. In our study, the expression level of *MyoG* was high at 70 and 90 dpc in TC pigs and 55 and 70 dpc in YK pigs, but obviously decreased after 90 dpc. *MYF6* was at a low level at 30 dpc, and then began to increase at 40 dpc in TC and YK pigs, which indicates that it may be involved in differentiation before and after birth. The *MEF2* and *MRF* families coordinate the regulation of muscle-specific genes and myogenic differentiation. *Mef2d* deletion in mice has little effect on skeletal muscle development, but deletion of *Mef2c* has been shown to cause defects in muscle formation [50]. Additionally, *Mef2c* and *Mef2d* also play a role in muscle regeneration of satellite cells [51]. *Mef2c* and *Mef2d* have similar expression patterns in TC and YK breeds, but *Mef2c* showed a higher expression level in our study. *Myostatin* (*MSTN*) is an inhibitor of skeletal muscle growth [52], and knockout mice show a dramatic increase in muscle mass [53]. Our data show high expression of *MSTN* in two phases, 40–55 dpc and 70–90 dpc, to inhibit over-proliferation of myoblasts when the primary and secondary fibers are forming. *MYOZ1* encodes a structural protein that constitutes the Z-lines in skeletal muscle. The expression of *MYOZ1* increases gradually with age in both breeds, but was higher in early TC embryos, and lower in later TC embryos and piglets, than in YK. *IGF2* is a critical regulatory factor for growth and differentiation of mammalian muscle and fetal development [54]; the sequencing results show high expression of *IGF2* across 40–105 dpc, and lower expression after birth in TC and YK pigs. This suggests that *IGF2* plays an important role during the key stages of primary and secondary fiber growth and development in the prenatal stage. Pathway analysis showed that *ENO3* is involved in the glycolysis biological process, and participates in energy metabolism for muscle development [55]. The *ENO3* expression level was higher in the postnatal stage than the prenatal stage in both breeds, but was higher in later YK embryos and piglets than in the TC breed. This indicates that *ENO3* is important for muscle development in the later period, and mainly provides energy by glycolytic metabolism.

**Figure 7:**
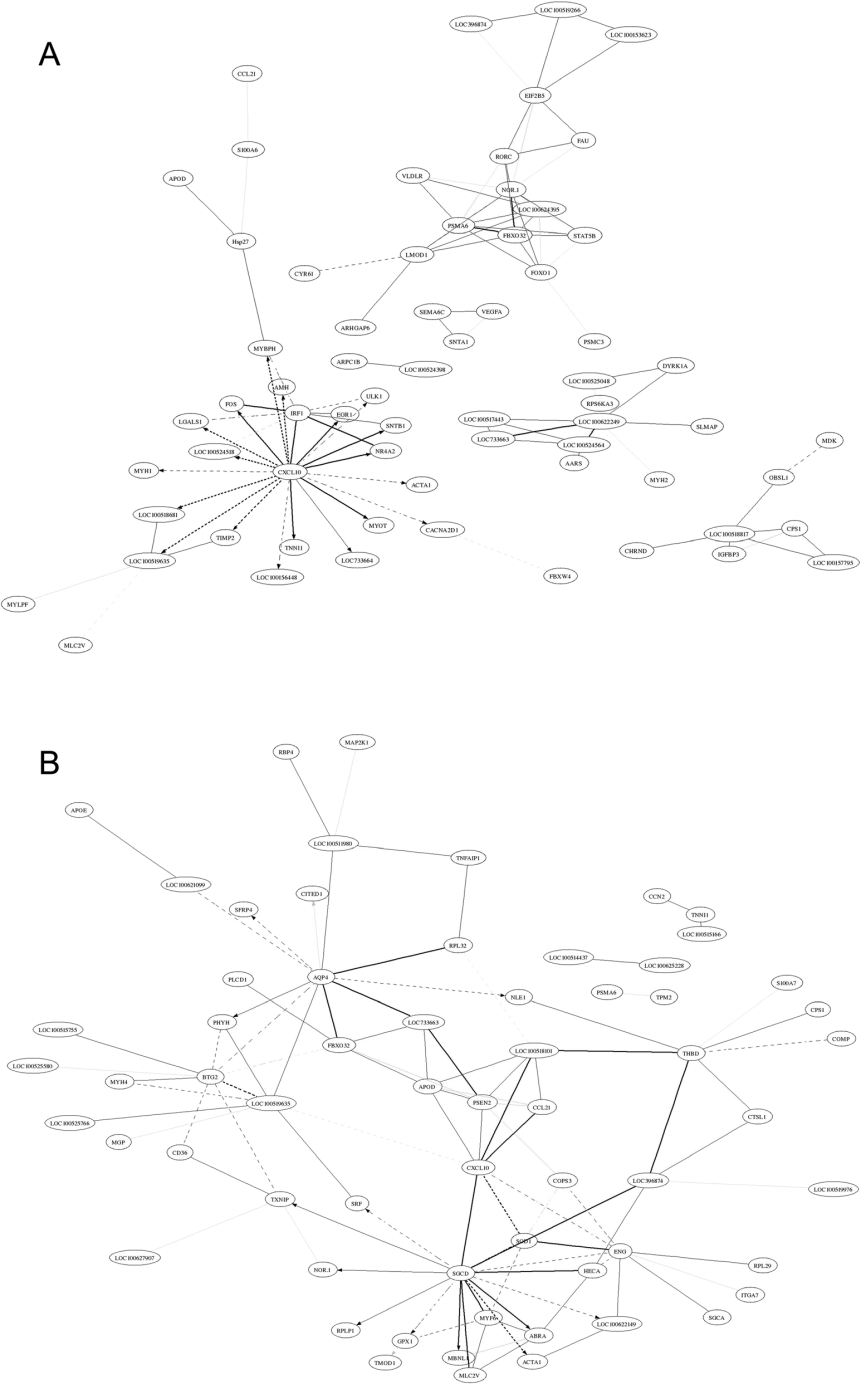
Correlation networks from muscle development related to genes between two breeds. **A**: Tongcheng pigs, **B**: Yorkshire pigs. The solid and dotted lines indicate positive and negative correlation coefficients, respectively; and the line intensity denotes their strength. Each straight arrow, from tail to head, indicates the interaction direction. The gene networks were obtained by the method “GeneNet” from the R package and display the top 100 largest absolute correlations.

GO terms were screened for significant enrichment according to functional, cellular, and biological annotation to investigate gene roles in the 11 stages of muscle development of TC and YK pigs. Many DE genes were focused on the features associated with contractile fiber, myofibril, actin cytoskeleton, sarcomere, contractile fiber part, and myosin complex (Additional file 6: Tables S3, Additional file 7: Table S4); the results show muscle-specific differential expression across muscle growth for these genes. Interestingly, many DE genes were also enriched for cellular components, which were distributed in the cytoplasm, cytoplasmic part, cellular, intracellular, and intracellular parts, which indicates that cellular signal transduction, intracellular communication, and transportation perform important roles in myogenesis. These annotations give valuable insights into the regulatory pathways for studying specific processes and biological functions in skeletal muscle growth. We also found that DE genes had different levels of enrichment between the distinct comparison libraries (Figure 4, Additional file 8: Figure S4); many DE genes were aligned with GO terms related to muscle characteristics and diseases. Therefore, we selected DE genes related to skeletal muscle development based on their GO annotation; 984 genes were obtained, 851 of these in TC pigs and 523 in YK pigs (Additional file 12: Table S8). This indicates a more complicated molecular regulatory mechanism for muscle development in TC pigs, and that more DE genes were involved in muscle fiber growth (Additional file 12: Table S8, Figure 1), which is consistent with LongSAGE sequencing results [23]. We constructed gene regulatory interaction networks with the top 100 correlated genes (Figure 7) to explore the key candidate genes related to muscle development. These potential gene interaction networks regulate the processes forming muscle fibers, and the interaction relationship is different between TC and YK pigs. The genes *CXCL10*, *EIF2B5*, *PSMA6*, *FBXO32*, and *LOC100622249* played vital roles in the muscle regulatory networks in the TC breed, while the genes *SGCD*, *ENG*, *THBD*, *CXCL10*, *AQP4*, and *BTG2* performed key roles in the interactions in YK pigs. We then carried out pathway analysis of the DE genes and a heat map of the significant pathways (Figure 5) to shed light on functional regulation. These analyses indicate that the pathway for Parkinson’s disease is significant (FDR <0.05) in both breeds. The pathways for Huntington’s disease and Alzheimer’s disease showed significant enrichment (FDR <0.05) in YK pigs but not in TC pigs; these may be critical target candidate genes for muscle diseases.

## Conclusions

We provide a new insight into transcriptional profiles of skeletal muscle development at 11 stages of TC and YK pigs using a genome-wide deep sequencing method. The critical stage for the formation of primary and secondary fibers is from 40 to 63 dpc for both Tongcheng and Yorkshire pigs. Muscle development in early TC embryos is more intense than in YK embryos, and is more intense in YK piglets than in TC piglets from the natal day to 1-week postnatum; this suggests that muscle fiber formation in TC pigs is initiated earlier than in YK pigs (30–40 dpc), while the muscle maturation processes are more complicated in early YK piglets (0–1 wpn). Many DE genes related to muscle development show phase-specific regulatory mechanism in myogenesis. Moreover, muscle contractile speeds and locomotion capacity are different in TC and YK pigs, which may be associated with more MyHC-I fibers in TC pigs and more MyHC-IIb fibers in YK pigs. The transition of energy metabolism between the oxidative phosphorylation and gluconeogenesis pathways affects the characteristics of the muscle fiber types. Furthermore, this study of skeletal muscle transcriptional profiles is helpful in understanding the differences between the genetic mechanisms in TC and YK pigs, and for future work exploring breed-specific changes in muscle development.

## Methods

### Ethics statement

The experimental animal procedures were followed in accordance with the approved protocols of Hubei Province, PR China for the Biological Studies Animal Care and Use Committee.

### Animals and muscle tissue preparation

Muscle tissue samples were obtained from 11 time points of skeletal muscle developmental stages for two breeds. Purebred TC and YK gilts (n = 40 per breed) were bred to purebred boars of their respective breed. Thirty-five gilts were slaughtered at each of seven time points for each breed (30, 40, 55, 63, 70, 90, and 105 dpc (days post coitus)) and the longissimus muscle tissue was rapidly dissected from each fetus (70 fetus samples). The remaining five gilts were allowed to farrow and longissimus muscle was collected from the piglets from each litter at 0, 1, 3, and 5 weeks of age (40 piglet samples). One hundred and ten muscle samples were taken and prepared. All samples were immediately snap frozen in liquid nitrogen and stored at −80°C until further use.

### RNA quality assessment, cDNA library construction, and sequencing

Total RNA was extracted from the 110 frozen longissimus muscle tissues using TRIzol reagent (Invitrogen, CA, USA) according to the manufacturer’s instructions. The RNA integrity and concentration were assessed using the Agilent 2100 Bioanalyzer (Agilent Technologies, Palo Alto, CA, USA) and met the experimental requirement of the Solexa sequencing platform. The quality of all the sample solutions had RNA integrity numbers (RIN) ≥ 7.0 and 28S/18S ≥ 1.0. RNA libraries were constructed and Solexa sequencing was performed by the Beijing Genomics Institute (BGI) on an Illumina Genome Analyzer. Sequencing tags were obtained using Illumina’s Digital Gene Expression Tag Profiling Kit according to the manufacturer’s instructions. These tags were aligned and identified based on two reference genomes, Ensembl *Sus scrofa* 10.2.68 and the UniGene database. Clean tags were obtained after filtering the adaptor tags, empty tags, low-quality tags, and one-copy tags from the total tags, and then they were aligned to the reference database and annotated.

### DE genes

Clean tags of each library were normalized by means of Tags per Million (TPM) to obtain normalized gene expression levels. DE genes were determined using the NOISeq-bio methods described by Tarazona et al. [56]. The fold changes (log_2_ ratio) were estimated according to the normalized gene expression level in each sample. We used the absolute value of log_2_ ratio > 1 and probability > 0.7 as the threshold to judge significant differences in gene expression.

### Cluster analysis

Similarities in gene expression patterns usually indicate that those genes have similar functions. The purpose of the clustering analysis was to screen the function of similar genes and cluster them together in the TC and YK pig breeds, thereby finding information on the function of the unknown genes, or unknown functions of known genes. We analyzed the systematic cluster of the two pig breed libraries using MEV software [57] for all DE genes in each breed.

### GO analysis

GO is suitable for gene functional classification for all types of prokaryotes and eukaryotes [58], and this was applied to analyze the main functions of the DE genes. A hypergeometric distribution method was used for the GO significant enrichment analysis function of the DE genes. All DE genes were aligned and mapped using the GO database (http://geneontology.org/) for TC and YK pigs, and then the significant enrichment terms and gene list of each term were obtained for both breeds. The GO analysis can be helpful in determining the main biological function of classification for the DE genes, and for exploring the molecular functions and biological processes. The false discovery rate (FDR) was calculated to correct the *P*-value for the GOs of all the DE genes.

### Assignment of DE genes to KEGG pathways

Pathway analysis helps to further understand the distinct functions of gene regulation and identify the significant pathways of the DE genes according to the KEGG database (Kyoto Encyclopedia of Genes and Genomes), which is a major public database on pathway enrichment analysis [59] (http://www.kegg.jp/kegg/pathway.html). The DE genes of TC and YK pigs were aligned to the KEGG database based on the entire genome background, which revealed any significant enrichment for DE genes in the pathway. A *P*-value and FDR was returned after pathway analysis for each breed. The threshold of significance was defined by FDR <0.05. Furthermore, a heat map of significant functional pathway enrichment was gained between the 10 comparison groups of each breed according to the FDR value using the Genesis software (http://genome.tugraz.at/genesisclient/genesisclient_description.shtml).

### Real-time qPCR

Five DE genes were chosen to verify the accuracy of the sequencing results, which was tested by real-time fluorescent qPCR on an ABI 7900 platform (Life Technologies, carlsbad, USA). qPCR was performed using SYBR Green I Real-Time PCR Master Mix (Toyobo, Osaka, Japan) according to the manufacturer’s instructions. All PCR reactions were carried out in triplicate. The samples used for real-time PCR assays were the same as those for the Solexa sequencing experiments. The RT-PCR reaction was carried out in a total reaction mixture of 20 μL as follows: 0.3 μL of F primer (10 mM), 0.3 μL of R primer (10 mM), 10 μL of SYBR Green Real time PCR Master Mix, 0.5 μL of cDNA, and 8.9 μL of double-distilled H_2_O. The RT-PCR was performed as follows: 94°C for 4 min, 34 cycles of 94°C for 30 s, 57°C for 30 s, and 72°C for 30 s, and a final extension at 72°C for 5 min. Expression levels of commonly used internal genes were inconsistent and showed variation across the 11 different developmental stages of skeletal muscle between the TC and YK libraries. Therefore, the relative expression level of mRNA was calculated using *RAD23B* as an endogenous reference gene using the 2^-ΔΔCt^ method. The Spearman’s correlation coefficient was further calculated for each gene using the normalized data to quantify the consistency between the Solexa sequencing and RT-qPCR experiments. Additional file 13: Table S9 lists the gene-specific primers for gene expression.

### Histology and histochemistry of 22 samples from muscle fibers

We applied the hematoxylin and eosin method [60] to obtain the histology and histochemistry of the 11 different developmental stages of skeletal muscle fibers between TC and YK pigs. Each muscle tissue sample was processed routinely for paraffin embedding, and sections were cut and stained with hematoxylin and eosin for the muscle tissue morphogenetic contrast analysis. Micrographs were taken with the BX53 (Olympus Corporation, Tokyo, Japan) digital camera system. The muscle tissue image was magnified 400 times.

### Supporting data information

The transcriptome raw data of Tongcheng and Yorkshire pigs were submitted to the Sequence Read Archive (SRA) of the National Center for Biotechnology Information (accession number: SRA234513).

## Additional files

Additional file 1: Figure S1. Morphological variations of skeletal muscle samples between TC and YK pigs during muscle fiber development and growth. TC indicates Tongcheng pigs; YK indicates Yorkshire pigs. Seven stages before birth, including 30, 40, 55, 63, 70, 90, and 105 dpc (days post-coitus), and four stages after birth, including 0, 1, 3, and 5 wpn (weeks post-natum). Additional file 2: Table S1. Solexa sequencing result data of 11 muscle development periods in each pig breed. The data were obtained after normalization and took the average of five samples at each stage. Additional file 3: Figure S2. Distribution of total and distinct clean tags of each stage in TC and YK pigs. Additional file 4: Figure S3. Assessment of Solexa sequencing quality. A: distribution of gene expression B: the relationship between library size and the number of genes identified. C: saturation analysis for sequencing library. D: distribution of tags. E: relationship between tag positions and number in gene. F: distribution of tag position in gene. Additional file 5: Table S2. Summary of all DE genes for different libraries in TC and YK pigs. Additional file 6: Table S3. GO annotation for DE genes of different comparison libraries of TC. Additional file 7: Table S4. GO annotation for DE genes of different comparison libraries of YK. Additional file 8: Figure S4. Significant GO term enrichment between TC and YK pigs. The comparison libraries: 90 vs 105 dpc, 105 dpc vs 0 wpn, 0 vs 1 wpn, 1vs 3 wpn, 3 vs 5 wpn. Additional file 9: Table S5. Pathways of all DE genes in TC and YK. Additional file 10: Table S6. Pathways of DE genes of different comparison libraries in TC. Additional file 11: Table S7. Pathways of DE genes of different comparison libraries in YK. Additional file 12: Table S8. DE genes related to muscle development in both breed. Additional file 13: Table S9. Primer information of the genes in real-time fluorescent qPCR testing.
