# Supplementary figures and images for: Dynamic transcriptome profiles of skeletal muscle tissue across 11 developmental stages for both Tongcheng and Yorkshire pigs
Source: BMC Genomics. 2015 May 12;16(1):377. doi: 10.1186/s12864-015-1580-7 (PMC4437458; doi:10.1186/s12864-015-1580-7)

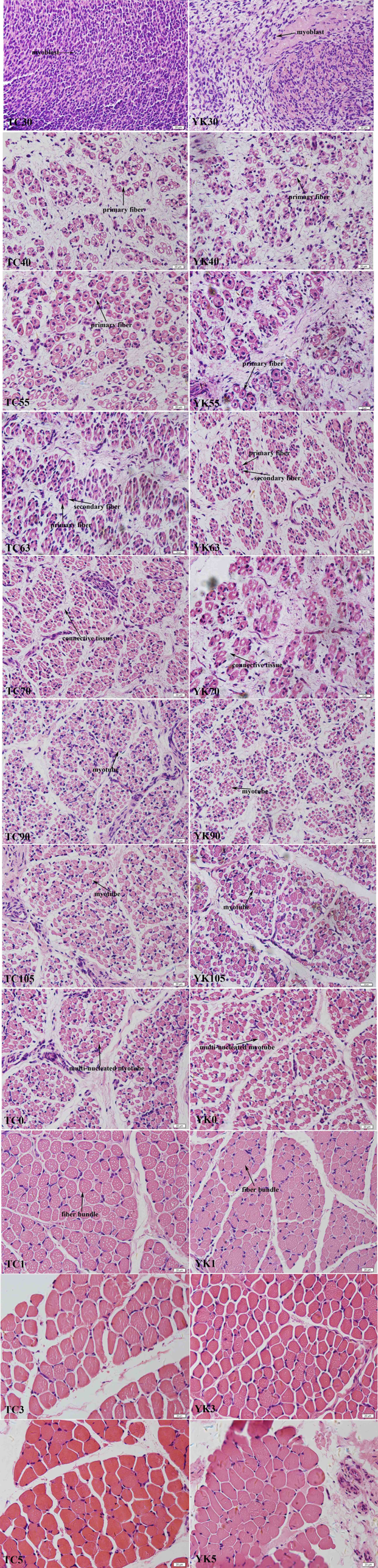

Supplement: Additional file 1: Figure S1. — Morphological variations of skeletal muscle samples between TC and YK pigs during muscle fiber development and growth. TC indicates Tongcheng pigs; YK indicates Yorkshire pigs. Seven stages before birth, including 30, 40, 55, 63, 70, 90, and 105 dpc (days post-coitus), and four stages after birth, including 0, 1, 3, and 5 wpn (weeks post-natum). [file 12864_2015_1580_MOESM1_ESM.jpeg]

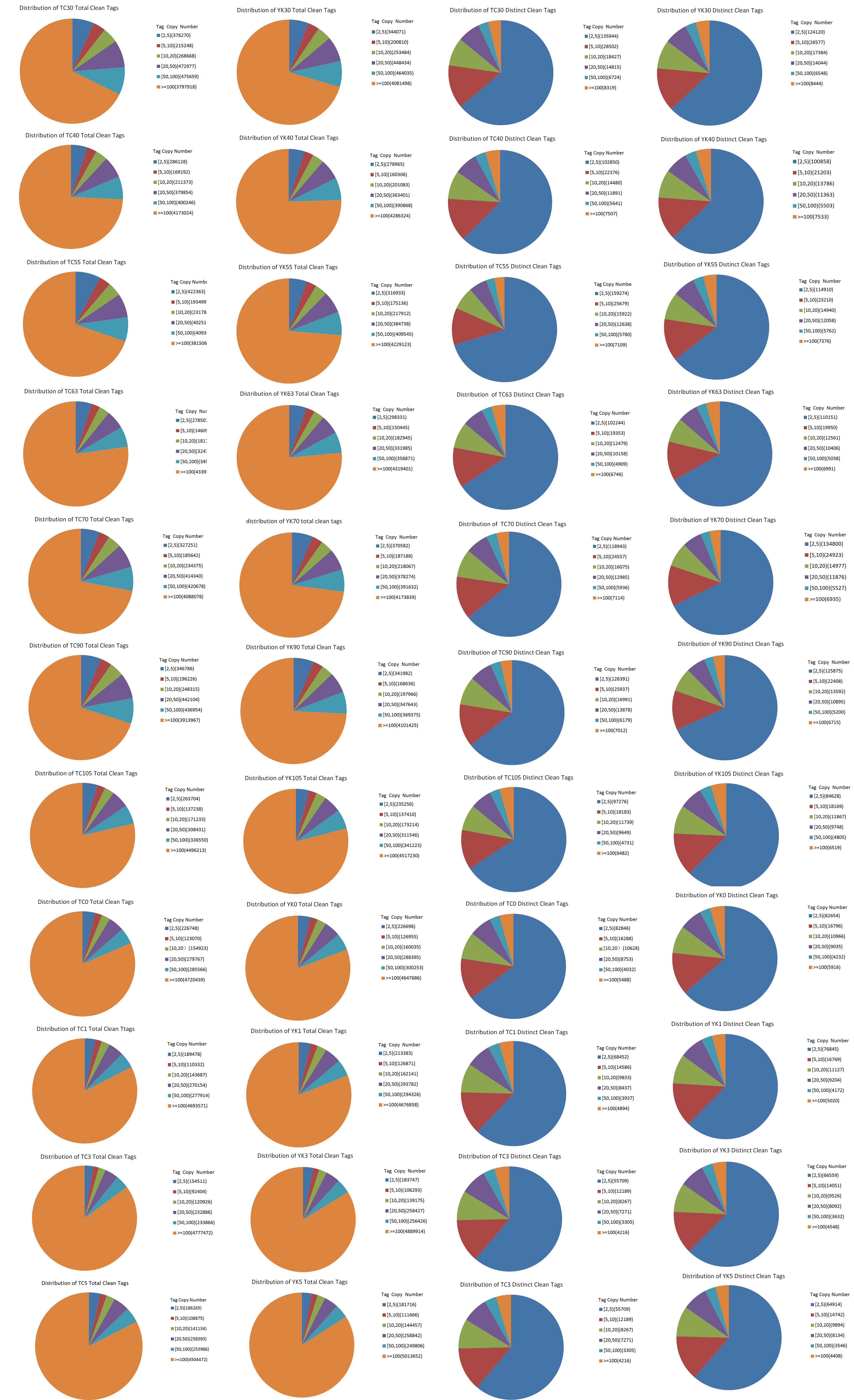

Supplement: Additional file 3: Figure S2. — Distribution of total and distinct clean tags of each stage in TC and YK pigs. [file 12864_2015_1580_MOESM3_ESM.jpeg]

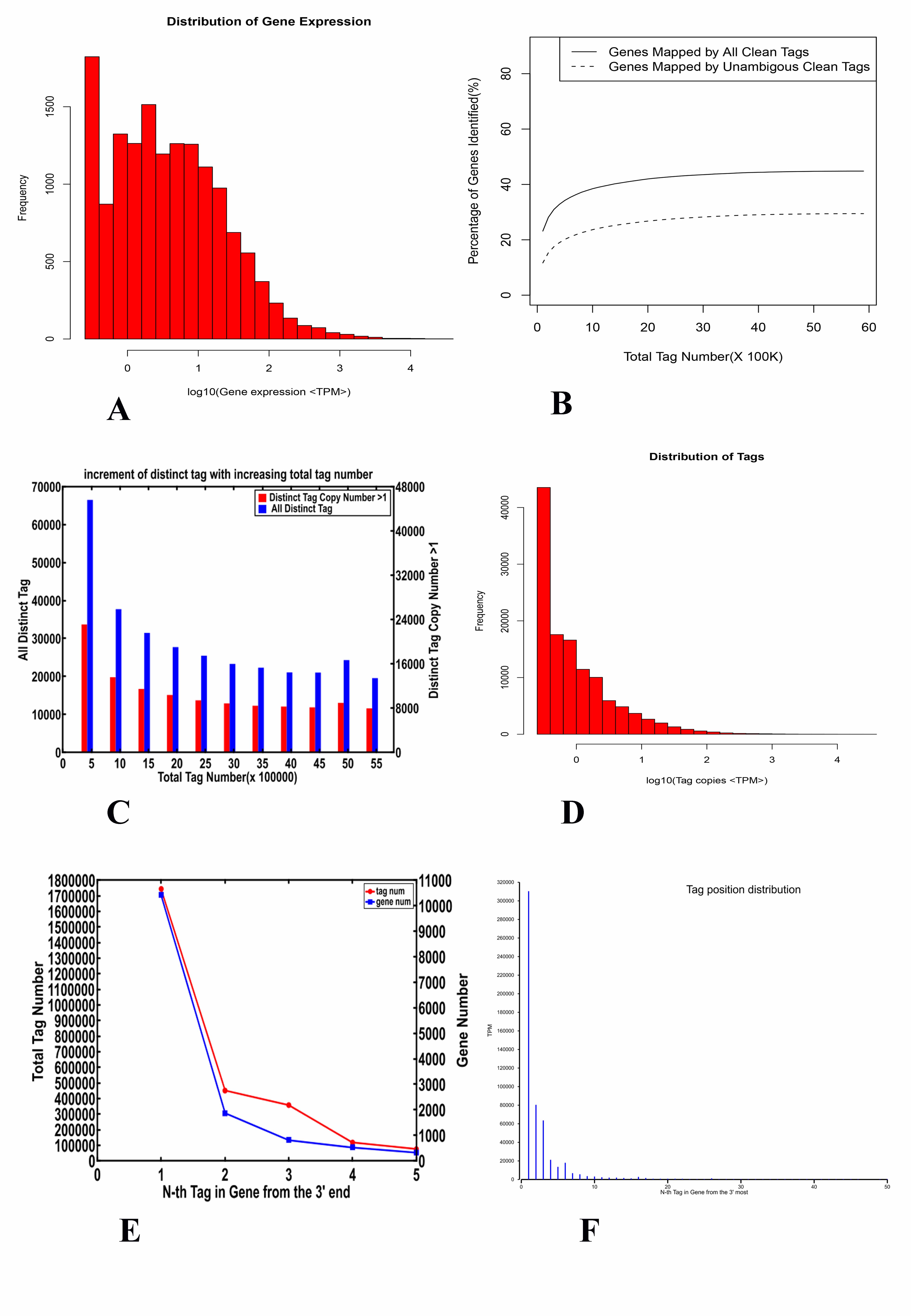

Supplement: Additional file 4: Figure S3. — Assessment of Solexa sequencing quality. A: distribution of gene expression B: the relationship between library size and the number of genes identified. C: saturation analysis for sequencing library. D: distribution of tags. E: relationship between tag positions and number in gene. F: distribution of tag position in gene. [file 12864_2015_1580_MOESM4_ESM.jpeg]

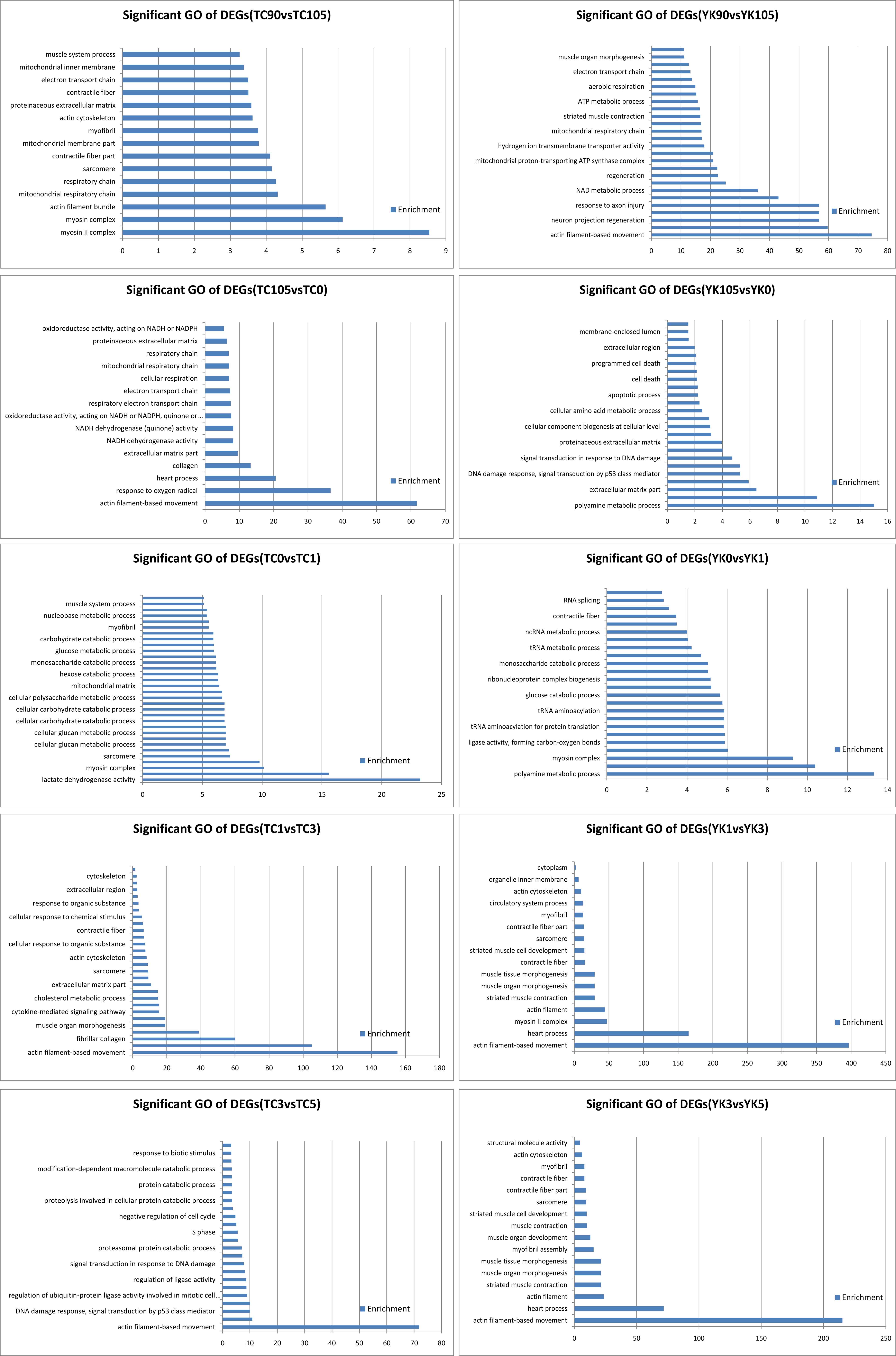

Supplement: Additional file 8: Figure S4. — Significant GO term enrichment between TC and YK pigs. The comparison libraries: 90 vs 105 dpc, 105 dpc vs 0 wpn, 0 vs 1 wpn, 1vs 3 wpn, 3 vs 5 wpn. [file 12864_2015_1580_MOESM8_ESM.jpeg]
